# Community outbreak of COVID-19 among people who use drugs attending a harm reduction centre in Barcelona, Spain

**DOI:** 10.1186/s12954-023-00804-y

**Published:** 2023-06-14

**Authors:** Nacho Sánchez-Valdivia, Maria Gabriela Barbaglia, Marc Olivella-Cirici, Noelia Girona Marcos, Mercè Gotsens, Amaia Garrido Albaina, Cristina Rius, Montse Bartroli, Gloria Pérez

**Affiliations:** 1 Agència de Salut Pública de Barcelona, Pl. de Lesseps, 1, 08023 Barcelona, Spain; 2 CIBER Epidemiología y Salud Pública (CIBERESP), Madrid, Spain; 3 Institut d’Investigació Biomèdica Sant Pau (IIB SANT PAU), Barcelona, Spain; 4 Universitat Pompeu Fabra, Barcelona, Spain; 5 Red de Investigación en Atención Primaria de Adicciones (RIAPAd), Madrid, Spain

**Keywords:** COVID-19, Outbreak, PWUC, Harm reduction, Inequalities, Vulnerable population

## Abstract

**Background:**

The COVID-19 pandemic particularly affected the health of vulnerable population, such as people who use drugs. Due to compromised baseline health status, certain drug uses and settings and socioeconomic deprivation related to poverty and homelessness, drug users faced higher risk of COVID-19 infection. They had difficulty in adhering to the public health measures (i.e. physical distancing, hand hygiene and mask use). Also, the struggle to implement non-pharmaceutical actions (i.e. test–trace–isolate–quarantine strategy) among SARS-COV-2-infected drug users and their close contacts challenged the public health response. Therefore, this study aimed to describe a community COVID-19 outbreak and its approach among drug users of a harm reduction programme in an outpatient drug treatment centre in Barcelona, Spain.

**Methods:**

We conducted an observational descriptive study of an outbreak of COVID-19 among people who use drugs attending the harm reduction programme of an outpatient drug treatment centre in the city of Barcelona, between July and October 2021 (*n* = 440). A passive search for cases was carried out with rapid antigens tests targeting symptomatic users who attended the facilities.

**Results:**

Nineteen positive COVID-19 cases were identified among symptomatic drug users, between July and October 2021, with an attack rate of 4.3%. Specific measures were taken to control the outbreak, such as offering accommodation to self-isolate in a low-threshold residential resource to homeless drug users who tested positive and intensifying the vaccination strategy. The management of the outbreak was carried out in close collaboration between the outpatient centre and the main public health stakeholders in the city of Barcelona.

**Conclusions:**

This study shows the complexity of managing and investigating COVID-19 outbreaks in vulnerable population groups. Epidemiological control measures, such as the test–trace–isolate–quarantine strategy, were challenging to implement due to technology-related barriers and socioeconomic vulnerabilities, especially homelessness. Community-based interventions, cooperation among stakeholders and housing-related policies were helpful in tackling outbreaks among people who use drugs. When addressing vulnerable and hidden populations, the perspective of inequalities should be included in epidemiological surveillance and outbreak control strategies.

## Background

The COVID-19 crisis affected millions of people globally [1]. The negative health effects of the pandemic were particularly strong among vulnerable populations. Focus was placed on older adults, people with chronic diseases or immunocompromised individuals and deprived communities [2]. However, other high-risk groups, such as people who use drugs (PWUD), were mainly neglected in institutional COVID-19 responses [3]; hence, efforts were made by local services, for example, in Barcelona, to effectively address the adverse consequences of the pandemic and facilitate self-isolation among vulnerable PWUD who were homeless [4, 5]. The pandemic highlighted the importance of considering social determinants of health in the management of health emergencies among vulnerable populations such as PWUD [6].

PWUD have a worse baseline health status [7, 8], as they usually could have compromised immune systems (e.g. about 18% of people who inject drugs live with HIV in the world [9]) and chronic respiratory diseases (e.g. people who use drugs have 86% increased odds of suffering chronic obstructive pulmonary disease) [10]. These pre-existing comorbidities, together with the high prevalence of smoking, are associated with a higher risk of SARS-COV-2 infection and more severe COVID-19 infection [8]. Certain drug use behaviours (i.e. sharing inhaling and/or injecting paraphernalia) and settings (i.e. poorly ventilated and small crowded rooms where drugs are consumed) also increase the possibility of contracting COVID-19 [8].

Many problematic drug users live in a situation of socioeconomic deprivation (i.e. poverty, unemployment, homelessness or poor housing, and food insecurity), which were aggravated during the COVID-19 crisis and increased their risk of infection [11–13]. For example, in Catalonia before the pandemic, about a 60% of PWUD obtain their main income from illegal sources and more than one in four live in the street [14]. In addition, this collective might be more affected by the disease because of delays in diagnosis and treatment due to unmet healthcare needs related to structural barriers to accessing healthcare services [8, 13, 15, 16]. Moreover, reductions and closures in some drug care services, such as harm reduction centres, during the first waves of the pandemic increased negative health impacts, due to difficulties in accessing sterile paraphernalia, opioid substitution treatment and/or basic healthcare [16–18].

In this context, vulnerable PWUD had difficulty in adhering to the public health measures of physical distancing, hand hygiene and mask use, since they usually congregate in tight spaces, with frequent interactions, and often share facilities [11, 13]. In addition, the struggle to implement the test–trace–isolate–quarantine (TTIQ) strategy among SARS-COV-2-infected patients and their close contacts, one of the main non-pharmaceutical actions to stop the spread of the virus, challenged the public health response [13].

Despite their many vulnerabilities, the impact of the COVID-19 pandemic in PWUD has hardly been explored, whereas there is evidence that this group was particularly affected by other epidemics, such as HIV and hepatitis [9, 19, 20]. Few studies have explored the impact of COVID-19 pandemic in PWUD [21], and to our knowledge no specific COVID-19 outbreaks in PWUD have been reported to date. Therefore, this study aimed to describe a community outbreak of COVID-19 and its approach among the users of a harm reduction programme in an outpatient addiction centre in the city of Barcelona, Spain, between July and October 2021.

### Outbreak detection

An outbreak was declared the 5th of August 2021 after the detection of an accumulation of five confirmed cases and one suspected case of SARS-COV-2 infection among users of the harm reduction programme of an outpatient addiction centre (Centre d’Atenció i Seguiment a les drogodependències (CAS), in Catalan) in the city of Barcelona, Spain. The centre, located in a socioeconomically deprived neighbourhood, provides harm reduction services, including a supervised drug consumption room for drug injection and inhalation. More information on Barcelona drug dependence care services can be found in Parés-Badell et al. [22].

The first case was symptomatic and tested positive on 29th July, 2021 on rapid antigens testing (RAT). The same day, the CAS notified the positive case to the competent authority, the Public Health Agency of Barcelona (Agència de Salut Pública de Barcelona (ASPB), in Catalan). The following week, the CAS identified and tested five more symptomatic cases. In this study, we report the epidemiological investigation of 19 cases reported up to 18th October, 2021.

The outbreak took place during the fifth wave of the COVID-19 pandemic (between June and October 2021), with a 14-day cumulative incidence rate of 1766 COVID-19 cases per 100,000 population (at the peak) in the city of Barcelona. During this phase, COVID-19 prevention measures in Catalonia (individual and general) were relaxed, although a night curfew had to be implemented to mitigate the impact of the increasing number of cases. The wave especially affected youth and young adults because the vaccination strategy had just begun for them, even though general COVID-19 vaccination levels were high (about 70% one dose coverage) [23].

The declaration and management of the outbreak was carried out by the ASPB programme in charge of the prevention, surveillance and control of COVID-19 in the city of Barcelona, in coordination with the Drug Dependence Prevention and Care Unit, which is the service that manages the outpatient drug centres of the ASPB.

## Methods

### Study design

We conducted an observational descriptive study of a community COVID-19 outbreak among PWUD attending the harm reduction programme of a CAS in the city of Barcelona, between July and October 2021 (*n* = 440). We assumed as population at risk those exposed PWUD who used the harm reduction programme services within the period of the outbreak (from 29th July to 19th October 2021).

### Case detection

**Fig. 1:**
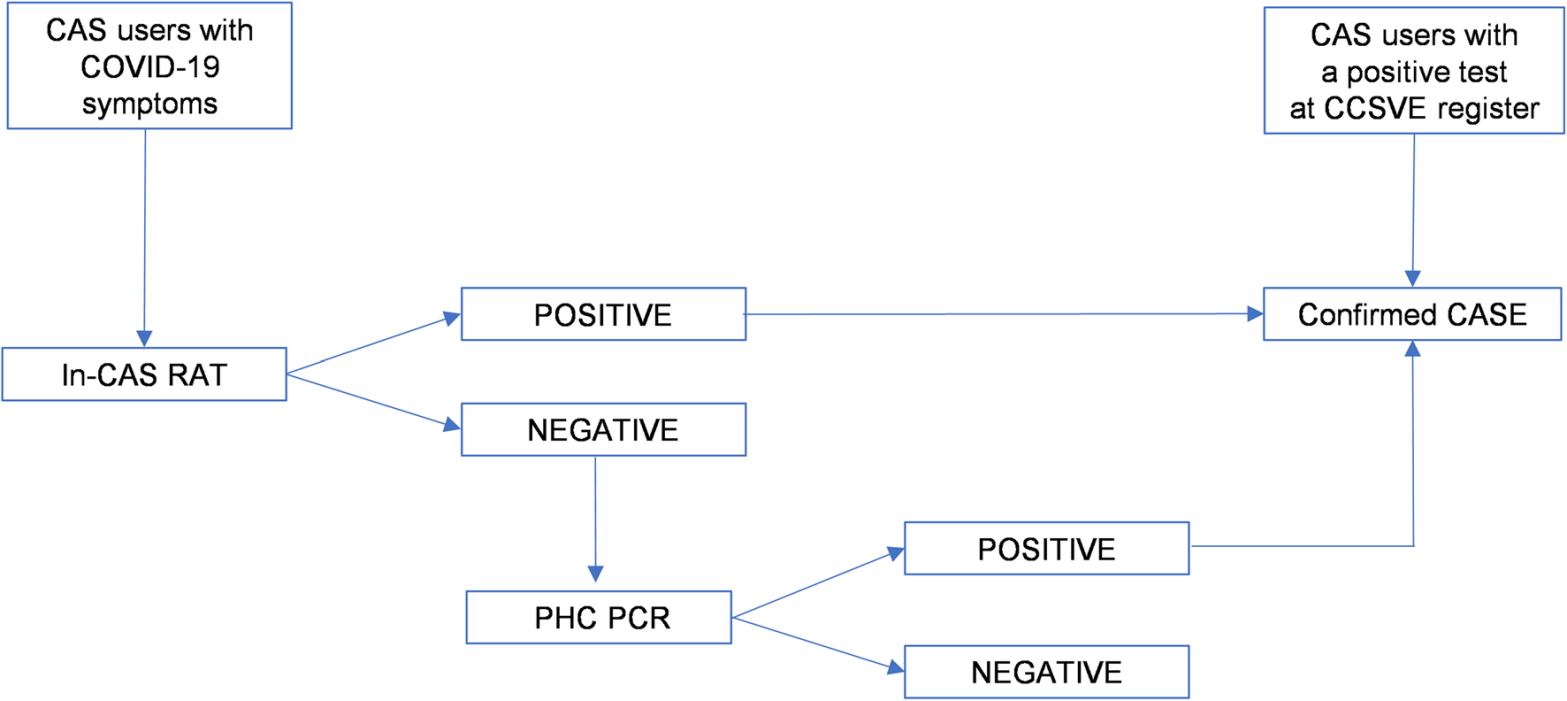
Flowchart of case detection and inclusion

A passive search for cases was carried out with RATs by the CAS nursing team targeting symptomatic users (*n* = 15) who attended the facilities (Fig. 1). If there was a negative result but COVID-19 compatible symptoms, individuals were referred to a nearby Primary Health Care (PHC) centre to confirm the result with a polymerase chain reaction (PCR) test (*N* = 3). Any person with a positive SARS-COV-2 PCR or RAT performed by an accredited laboratory during the outbreak was included in the epidemiological investigation. Sequencing of the virus was not possible because most persons (*n* = 15) were tested using a RAT.

### Case definition

Cases were defined in accordance with the Catalan Health Department COVID-19 procedure in effect between August and October 2021 [24]. A confirmed case of COVID-19 was any user of the centre with a positive SARS-COV-2 PCR or RAT with a history of exposure to the harm reduction facilities from 2 to 14 days before symptom onset or time of testing, and who had no previous epidemiologic links with COVID-19 cases outside of the centre. A probable case was any user with sudden-onset respiratory illness who had not undergone PCR testing for SARS-CoV-2 or who had a negative RAT, with a history of exposure to the CAS from 2 to 14 days before symptom onset, and who had no previous epidemiologic links with COVID-19 cases outside of the centre.

### Contact study

All PWUD attending the harm reduction programme in the CAS during the study period were included as the population at risk and were considered as potential contacts (*N* = 440). We defined exposure as either accessing the injecting and/or inhaling rooms or using the showers, or the syringe exchange area, or the area offering heat and coffee. We were unable to carry out an in-depth close contact investigation because of difficulties in contacting cases by telephone and in identifying close contacts, as many of the cases shared crowded spaces with unknown people outside the CAS.

### Data collection

Positive cases were obtained from the COVID-19 registry of cases and contacts (CCSVE), and data on PWUD attending the centre were obtained from the harm reduction information system, both owned by the Health Department of Catalonia. We used the COVID-19 registry to obtain epidemiological variables related to infection (date of symptom onset, date of COVID-19 diagnosis, COVID-19 vaccination status and type of vaccine) and the harm reduction registry to obtain sociodemographic variables (age, sex and country of birth) and clinical variables related to drug use (routes of administration, main drug used, being in treatment, including methadone). Personal data were anonymized.

## Results

### Outbreak control measures

After the worldwide COVID-19 outbreak, the General Sub-Directorate of Drug Addiction of the Catalan Health Department adapted the general recommendations to protect the PWUD community and users of harm reduction programmes from COVID-19 infection [24]. All complementary considerations can be found here [24]. On the basis of these recommendations, the ASPB together with the CAS and the area PHC centre designed a protocol to manage COVID-19-positive cases among users of the CAS, with special focus on those who were homeless. The plan included actions on how to proceed when a user tested positive (or was suspected to be positive), how to identify, trace and follow-up close contacts and whom to notify about these cases, as well as hygiene prevention (e.g. mandatory mask use) and capacity measures inside the facility and adjusted operating hours (due to curfew and/or lockdown). The clinical follow-up was done by the CAS and PHC teams.

When the outbreak was declared, several measures were taken jointly between the ASPB services involved and the CAS to control the spread of infection among users of the harm reduction programme (Table 1).

**Table 1:** Measures to prevent and control the community outbreak of COVID-19 among users of the harm reduction programme of the CAS

Target	Measures
CAS professionals	(i) Increase prevention measures among those who were in direct contact with users suspected of having COVID-19 within the CAS facilities (e.g. use of FFP2 and surgical masks) (ii) Offer information about PPE placement and use (iii) Foster vaccination in coordination with the area PHC
All users	(i) Free daily new surgical masks (ii) Foster vaccination in coordination with the area PHC (iii) Capacity limitation in consumption rooms
Symptomatic users	(i) RAT to confirm diagnosis. If the person tested negative but still had symptoms, PCR test (ii) 14-day ban on accessing the CAS facilities (iii) Offer free-of-pay municipal shelter to isolate (iv) Follow-up of cases
Contacts	If vaccinated: (i) Obligatory mask use in the CAS (ii) Access permitted only to venues where masks could be kept (ban to inhalation supervised room, shower and eating rooms)
	If unvaccinated: (i) 10-day ban on access to the CAS facilities

Those positive cases who were homeless were offered the possibility of isolation in the municipal shelter for homeless PWUD, which had five places for COVID-19-positive hosts. This resource was free-of-pay and opened at the beginning of the pandemic in a joint initiative between the ASPB and the Social Rights area of the Barcelona City Council to provide asylum to homeless PWUD during the lockdown period mandated by the Spanish Government. This low-threshold residential centre for active PWUD provided harm reduction services combining psyco-social support and accommodation [4]. The facilities had limited capacity, which enabled them to host only COVID-19-positive persons, but not their close contacts.

Once the outbreak was declared, specific actions to control transmission, were taken among users and among workers. Suspicious cases were identified when they showed symptoms compatible with COVID-19, and a RAT was performed to confirm the infection. If they tested positive, they could not access the CAS for the subsequent 14 days. If they were homeless, they were offered the possibility of isolation in the municipal shelter. Users identified as potential contacts who were not correctly vaccinated were banned from accessing CAS facilities for 10 days. If they were vaccinated, users could use only those facilities where masks could be kept. However, any user could continue receiving psyco-educational, social and health care from the CAS street educators and participate in the syringe exchange programme. The identification of these contacts was determined by the harm reduction centre professionals with the support of the ASPB.

Furthermore, the CAS offered new surgical masks to all users daily. The vaccination campaign against COVID-19 with the single-dose Janssen vaccine among PWUD was reinforced during the period of the outbreak, in collaboration with PHC, which facilitated the vials. Vaccine doses were administrated by the nursing team of the harm reduction programme. Control protection measures among workers were also reinforced. We established the use of FFP2 masks for all workers, increased the use of personal prevention equipment (PPE) among those who were in direct contact with suspicious cases within the CAS facilities (for example, when they did RAT testing in-house with symptomatic users), and offered information about the correct use of PPE.

### Descriptive analyses

**Fig. 2:**
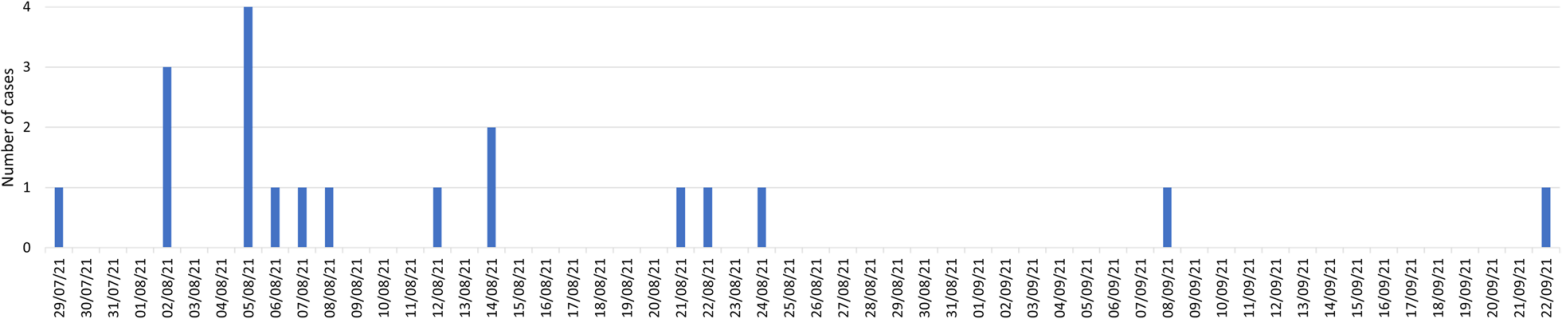
Epidemic curve of cases of COVID-19 by symptom onset or diagnosis date

The outbreak investigation was active for 82 days, from the positive result of the index case on 29th July until 19th October 2021, 28 days after the last case. The epidemic curve of cases is shown in Fig. 2.

A total of 19 positive cases were identified following a strategy of testing symptomatic PWUD, even though 89% (*n* = 17) had symptoms (Table 2). This is explained because two of the cases had partners among CAS users and were tested as easily traceable close contacts. The mean age of the cases was 40 years (SD: 10.0), and seven (36.8%) were women. Most of the positive cases (72.2%) were born outside Spain. The attack rate of the outbreak was 4.3% (19/440). Among the cases, 37% were vaccinated with the single-dose authorized Janssen vaccine. At that time, a single shot of the Janssen vaccine was considered sufficient to correctly vaccinate an individual by the national and European health authorities. More than half of the positive cases (10) self-isolated in the municipal shelter for homeless people actively using drugs. Regarding routes of drug administration among positive PWUD, four used both the inhalation and injection consumption rooms, six used only inhalation spaces, and five used only injection rooms. Data from four positive individuals were missing.

**Table 2:** Descriptive table with the characteristics of positive cases of the outbreak

Characteristics	Positive cases (*n* = 19*)	
	*N*	%
Sex		
Men	12	63.2
Women	7	36.8
Age (mean, SD)	40	10
Country of birth**		
Spain	5	27.8
Outside Spain	13	72.2
Symptomatic		
Yes	17	89
No	2	11
Type of test*		
RAT	15	83.3
PCR	3	16.7
COVID-19 vaccination status		
Correctly vaccinated***	7	36.8
Unvaccinated	12	63.2
Routes of drug consumption****		
Inhalated and injected	4	27
Inhalated	6	38
Injected	5	33
Municipal shelter use for isolation	10	53
Public health contacted	15	78.9
Public health interviewed*****	1	5

*1 case was considered probable due to COVID-19 compatible symptoms but negative test

**Data missing for a positive case

***Vaccinated with a single-dose Janssen vaccine

****Data missing for four positive cases

*****The interview consisted of an epidemiological survey and tracing of close contacts

More than three quarters of the cases in this outbreak (79%) were contacted by the ASPB COVID-19 surveillance team, although only one epidemiological survey and close contact interview could be carried out.

## Discussion

COVID-19 outbreaks in underserved, vulnerable and hidden populations such as PWUD are complex to investigate and manage. Despite the low attack rate of the reported outbreak (4.3%), non-pharmaceutical control measures such as the TTIQ strategy among cases and close contacts in PWUD were challenging to implement. To increase adherence to these measures, local and community interventions such as offering a free-of-pay shelter to PWUD with housing precariousness to complete self-isolation and working closely with the harm reduction programmes and PHC were helpful. In general, public health plans to tackle the COVID crisis needed an equity and specific approach based on social determinants of health in communities with a low socioeconomic level and housing precariousness, hampering adherence to measures, such as self-isolating when testing positive [5].

The low incidence of COVID-19 reported in this study could be partially explained by the special measures adopted by the outpatient addiction centres in Catalonia, and especially in the city of Barcelona, to both ensure the functioning of services for PWUD and the prevention of COVID-19 infections [24]. In Barcelona and some other Spanish cities, harm reduction centres continued offering services to PWUD throughout the COVID period (including the general lockdown), adjusting their operating hours (due to curfew) and their functioning (due to limitations on room capacity, mask use indoors, etc.) [17]. In other countries, such as Canada, drug-related services closed during the COVID pandemic and users faced a higher risk of adverse consequences (e.g. overdoses, substance use disorder, treatment disruption and other infections) [3, 8]. Moreover, the pre-existing model in Barcelona of integrating harm reduction and treatment into care centres for substance use could have buffered the impact of the health crisis on PWUD, as it maximizes access to harm reduction services and eliminates treatment barriers [21]. These facilities are often the only point of contact for vulnerable PWUD to access basic public services, such as health, health education and social security. Policies adopted in relation to the closure of drug services should be evaluated from a cost/benefit and inequalities perspective to avoid provoking further suffering and burden on a clearly vulnerable population in the future.

Many of the users of the harm reduction programme (about 40%) had socioeconomic vulnerabilities, including homelessness or extremely precarious housing conditions (e.g. living in the street or in huts in city parks), which hampered control within the community. Some studies have reported that hidden populations without access to adequate housing (such as those living in overcrowded conditions) are at an increased risk of COVID-19 and related complications [25]. Additionally, gender has been associated with differentiated trajectories through drug use and also homelessness [26].

In this respect, having a low-threshold residential resource for homeless people using drugs in Barcelona with some places for COVID-19 positive cases helped some PWUD to complete self-isolation. During the investigation and management of the outbreak, 10 cases used this service offered by the City Council, thus avoiding contact between positive cases and other non-COVID-19 users of the harm reduction centre, which may have helped to control the spread of the virus among the PWUD community. Similar initiatives were implemented in Lisbon (Portugal) [27] and Ontario [15]. Furthermore, the municipal shelter for homeless people using drugs in Barcelona adopted a gender perspective into its structure resources (i.e. offering safe accommodation and incorporating a gender-perspective approach normative), services and activities (i.e. considering non-mixed spaces, organizing gender-specific activities and offering trauma-informed care) and human resources (i.e. having a gender referent and external supervision) [4].

Additionally, vaccination against COVID-19 might have been an important strategy to reduce the burden of infection in drug users, which was aggravated due to their socioeconomic vulnerability, drug use and setting [28]. In our case, the vaccination campaign was reinforced during the outbreak with visuals and posters in several common areas of the CAS (i.e. consumption rooms, nursing room, café and rest area) and the vaccine was personally offered to all users when accessing the facilities. Indeed, they could get inoculated in the same facility by the nursing team of the harm reduction programme or in the PHC centre located near the CAS.

Nevertheless, we also found some challenges in tackling the epidemiological investigation and control of this community COVID-19 outbreak. The TTIQ strategy was difficult to implement among PWUD in the harm reduction centre due to telephone technology unavailability, since it involves calls and the use of smartphone apps. The lack of mobile phones was a critical barrier to contacting positive cases in this community, collecting clinical information on the disease (e.g. symptoms and date of symptoms onset) and obtaining data on transmission chains (e.g. close contacts register and venues attended in the transmission risk period). Eventually, most of the epidemiological surveys could not be carried out. Technology-based strategies have been identified as barriers for some PWUD, because of the lack of access to telephones and inadequate internet connection [29]. Another reason was the reluctance among PWUD to be interviewed by public health services, due to privacy concerns, mistrust and the fear of stigmatization [30]. Being asked about their personal and close contacts for contact tracing could have triggered rejection of telephone calls from the surveillance team.

These difficulties, coupled with the fact that many positive cases attended small and crowded spaces and had unknown interpersonal contacts, constrained our ability to trace close contacts and perform tests, hampering comprehensive epidemiological investigation and control of the outbreak [31]. Indeed, these limitations, together with testing only COVID-19 symptomatic cases, led to underdiagnosis of asymptomatic cases and the impossibility of establishing chains of transmission among the identified positive cases within the outbreak investigation, as cases could not be contacted by public health services in charge of epidemiological inquiries. Improving the notification system and adherence to TTIQ strategy among PWUD and specifically including PWUD vulnerabilities in public health strategies could help to contain future outbreaks, and save valuable healthcare resources [7, 32].

Despite these obstacles, the existing community engagement with the users attending the harm reduction programme was particularly important in applying preventive public health measures (for example, developing and executing protocols against COVID) and also implementing contingency interventions to halt COVID-19 transmission chains [33], as was evident in the outbreak reported here. In addition, the cooperation between different public health stakeholders (ASPB, PHC and CAS) in the management of the outbreak, as well as in its prevention and surveillance, and the intersectoral approaches, emerged as key elements to overcome the difficulties related to the socioeconomic context of PWUD and the lack of specific resources and research on this collective [19]. For example, in Barcelona, as explained, during the COVID-19 crisis, the harm reduction centre joined forces with the PHC in the area to identify positive cases and facilitate vaccination among this group. Another example is the launch of the municipal shelter with the City Council for homeless PWUD [4]. Reinforcing collaboration between agents and departments and fostering community-based approaches would facilitate disease outbreak management in the future and, eventually, would protect vulnerable communities.

## Conclusions

All in all, epidemiological surveillance and control strategies of COVID-19, such as TTIQ, must include the inequalities perspective to effectively tackle outbreaks among vulnerable and hidden populations, such as drug users, and consider disease prevention and control in relation to their socioeconomic needs [7]. Interventions in relation to homelessness, community building and engagement and intersectoral cooperation would help to implement public health measures. Therefore, more solutions and resources should be allocated to effectively adapt public health crisis approaches in order to ensure correct management of outbreaks among PWUD.

## Data Availability

Not applicable due to privacy concerns.

## Abbreviations

ASPB: Agència de Salut Pública de Barcelona
CAS: Centre d’Atenció i Seguiment a les drogodependències
CCSVE: COVID-19 registry of cases and contacts
PCR: Polymerase chain reaction
PHC: Primary health care
PPE: Personal protective equipment
PWUD: People who use drugs
RAT: Rapid antigens test
TTIQ: Test–trace–isolate–quarantine
HIV: Human immunodeficiency virus
